# Correction: Bayesian inference of kinetic schemes for ion channels by Kalman filtering

**DOI:** 10.7554/eLife.96697

**Published:** 2024-02-09

**Authors:** Jan L Münch, Fabian Paul, Ralf Schmauder, Klaus Benndorf

**Keywords:** Human

Münch JL, Paul F, Schmauder R, Benndorf K. 2022. Bayesian inference of kinetic schemes for ion channels by Kalman filtering. *eLife* **11**:e62714. doi: 10.7554/eLife.62714.

It has come to our attention that a definition is lacking in the main text and subsequently some equations are not written consistently. We further noted that a text fragment is incorrect, the subpanel upper-scale legend in figure 3b is missing and indices are not always used consistently throughout the paper.

In detail:

**(1) The following definition needs to be added to the main text:**

Corrected text:

‘The set of all measurements up to time t is defined by $Y_{t}=\left\{y_{1},\ldots ,\;y_{t}\right\}$ . If the system strictly obeys Equation 3 and Equation 4 then the KF is optimal in the sense that it is the minimum variance filter of…’

Original text:

“If the system strictly obeys Equation 3 and Equation 4 then the KF is optimal in the sense that it is the minimum variance filter of...”

**(2) Based on this definition, we can write Equation 5–Equation 8 more consistently to represent the recursive nature of the algorithm. The way these equations are used remains exactly the same.**

Corrected Eq. 5:

$$P(n_{t}|Y_{t-1})=\int P(n_{t}|n_{t-1})P(n_{t-1}|Y_{t-1})\;dn_{t-1}\text{,}$$

Original Eq. 5:

$$P(n_{t})=\int P(n_{t}|n_{t-1})P(n_{t-1}|y_{t-1})\;dn_{t-1}\text{,}$$

(The left-hand side omitted the conditioning on $Y_{t-1}$ on the right-hand side.)

Corrected Eq. 6:

$$P(n_{t}|Y_{t-1})=\int N(n_{t}|Tn_{t-1},Q_{t-1})P(n_{t-1}|Y_{t-1})\;dn_{t-1}$$

Original Eq. 6:

$$P(n_{t})=\int N(n_{t}|Tn_{t-1},Q_{t-1})P(n_{t-1}|y_{t-1})\;dn_{t-1}$$

(The left-hand side omitted the conditioning on $Y_{t-1}$ on the right-hand side.)

Corrected Eq. 7:

$$P(n_{t}|Y_{t})=\frac{O(y_{t}|n_{t})P(n_{t}|Y_{t-1})}{\int O(y_{t}|n_{t})P(n_{t}|Y_{t-1})\;dn_{t}}\text{,}$$

Original Eq. 7:

$$P(n_{t}|y_{t})=\frac{O(y_{t}|n_{t})P(n_{t})}{\int O(y_{t}|n_{t})P(n_{t})\;dn_{t}}\text{,}$$

Corrected Eq.8:

$$P(n_{t}|Y_{t})=\frac{N(y_{t}|Hn_{t},\Sigma_{t})P(n_{t}|Y_{t-1})}{\int N(y_{t}|Hn_{t},\Sigma_{t})P(n_{t}|Y_{t-1})\;dn_{t}}\text{,}$$

Original Eq. 8:

$$P(n_{t}|y_{t})=\frac{N(y_{t}|Hn_{t},\Sigma_{t})P(n_{t})}{\int N(y_{t}|Hn_{t},\Sigma_{t})P(n_{t})\;dn_{t}}\text{,}$$

**(3) A sentence on page 9 below Eq. 15 was scrambled:**

Corrected Text:

“…performs consistently poorer. It may seem surprising that even for $\sigma_{op}/i<0.01$ the two stochastic algorithms start to produce different results. But considering the scaling (Materials and...”

Original text:

“…performs consistently poorer. It may seem surprising that even for In fact, the KF method beha the two stochastic algorithms start to produce different results. But considering the scaling (Materials and...”

**(4) In Figure 3b, the legend of the upper scale needs to be added:**

Corrected Figure 3 is shown here:

**Figure 1:**
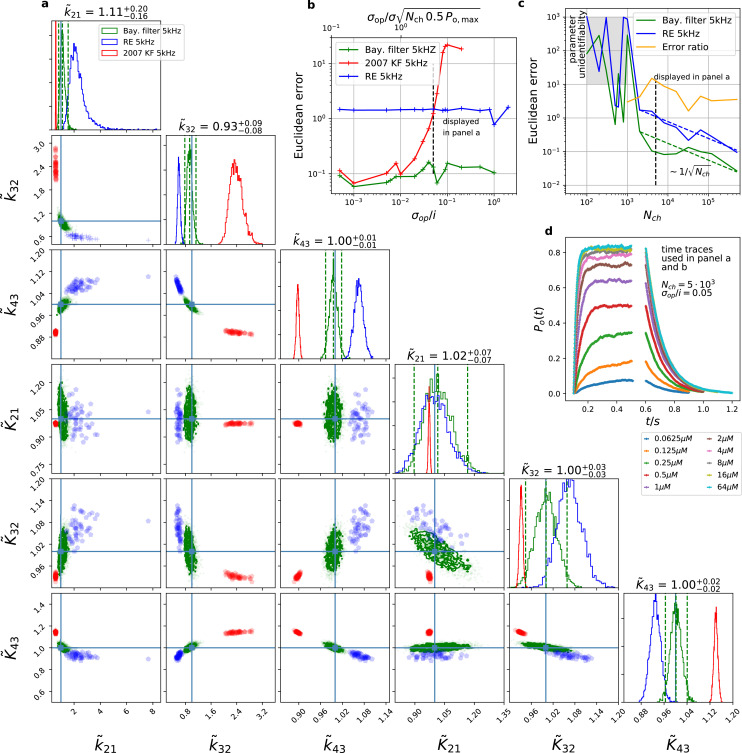


The originally published Figure 3 is shown for reference:

**Figure 2:**
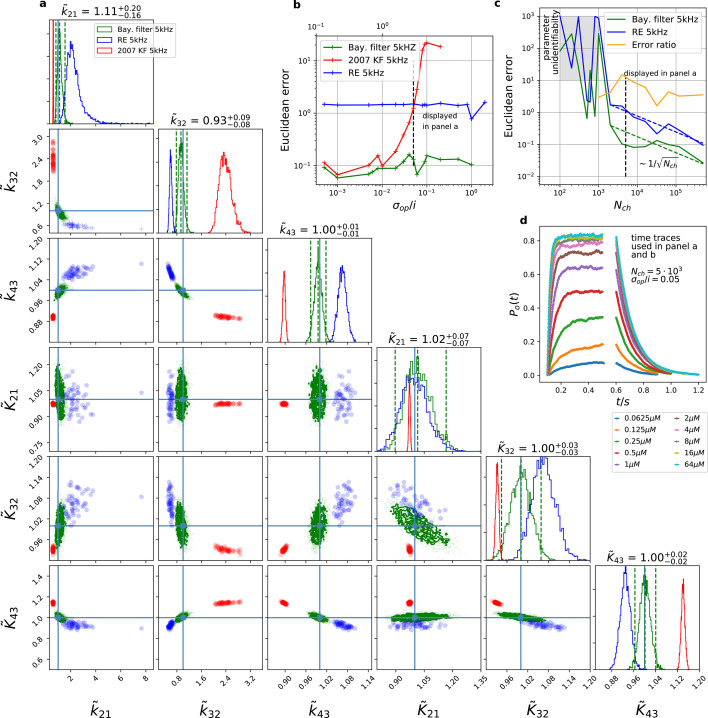


(**5) We were inconsistent in the use of indexes for variance and standard deviations. E.g. for measuring noise: both** $\sigma_{ex}$ **and** $\sigma_{m}$ **was used. Changes in two lines in Table 1 address this issue:**

Corrected line 16, first column:

“ $\sigma_{m}^{2}$ and $\sigma_{ex}^{2}$ ”

Original line 16, first column:

“ $\sigma_{m}^{2}$ ”

Corrected line 20, first column:

“ $\sigma_{back}^{2}$ ”

Original line 20, first column:

“ $\sigma_{bulk}^{2}$ ”

The article has been corrected accordingly.

