# Supplementary material for: Bayesian inference of kinetic schemes for ion channels by Kalman filtering
Source: eLife. 2022 May 4;11:e62714. doi: 10.7554/eLife.62714 (PMC9342998; doi:10.7554/eLife.62714)
Supplement: Source code 1. [file elife-62714-code1.zip › MakroSim/Quick Start Guide for MakroSimV46uint32.pdf]

Quick Start Guide for MakroSimV46uint32

General

This software is modeling the behavior of ion channels in a patch by a given markov-model. We get the simulation results of the open probability Po(t) and the binding degree Bi(t) for a patch with the number N of ion channels. For a number of k repetitions of the simulation the program delivers the mean of Po and Bi and their sem. Thus Po an Bi show the typically channel noise. Not included is the experimental noise and the convolution by the sampling function, as we consider the macroscopic current and the fluorescence signal.

What to do

You need in the current folder the files MakroSimV46uint32.m and MakroSimV46uint32.fig, and also one model-file and one pulse-file. Fulfill MakroSimV46uint32.m in the Command Window of Matlab and press „enter" – the program will start.
Select in the menu-bar the menu **Model** an open the m-file of the desired model. (Model-files, created with our GUI-fitprogram, could also load as mat-files). Then use the menu **Pulse** to open the m-file for the desired pulse. It will appear the file-names of the selected model and pulse and the constant timestep **dt**, fixed in the pulse-file.
You can set the number of channels in the patch (default N=100) and the number of repetitions (default k=1) of the simulation. Pressing „**Start**" starts the simulation and you can see the progress in the counter. By using the „**Stop**"-button the simulation can be aborted at any time.

The default simulation-method is based on „**dwell time simulation**", where after an exponential distributed dwell time the next neighboring state will be selected arbitrary according the given transition probabilities. If the dwell time is much shorter than the time step dt, this method will be not very suitable. The best way would be to shrink the time step dt. But in some cases, also the method „**calculate each time point**" delivers useful results. Here the probabilities are used to delay after the time dt or to change to any other state. However, fast transitions within dt are also not recorded. The radio button "**only theoretical curves**" shows the ideal course i.e. N tends to infinity.

By activating the checkbox "**add theoretical curve**" you get the ideal courses supplementary to the simulated courses in the figure. The checkbox "**do not erase old curves**" allows to maintain all the old courses in the figure, and every new curve gets a new color. By activating the checkbox "**consider diffusion effects**" the concentration jumps are taken into account and are described by exponentials according to c=c0*(1-exp(t/tau)). This function is approximated by ten sub-pulses with a constant concentration, whereby the variables tau are stored in the pulse-file for each jump.
With the button "**close**" you can finish the application. You will be asked if there is anything left to save. You can now save all simulated and theoretical data individually or all together either as an EXCEL- or as a csv-file. Of course, you can also do this after each simulation.

Wish you good success !

Questions welcome to: e.schulz@hs-sm.de

Eckhard Schulz[1] : development, mathematics, programming
Katharina Anhalt[1] : programming, special solutions in GU
Thomas Eick[2] : testing, working, adjustment

[1] Schmalkalden University of Applied Sciences, Faculty of Electrical Engineering, Blechhammer, 98574 Schmalkalden, Germany
[2] Institute of Physiology II, Jena University Hospital, Friedrich Schiller University Jena, 07743 Jena, Germany
