# Supplementary figures and images for: Bayesian inference of kinetic schemes for ion channels by Kalman filtering
Source: eLife. 2022 May 4;11:e62714. doi: 10.7554/eLife.62714 (PMC9342998; doi:10.7554/eLife.62714)

# Modell\_3C1O\_Jan

Ratekonstanten (Parameter)  
in  $\mu\text{M}^{-1}\text{ms}^{-1}$  bzw.  $\text{ms}^{-1}$

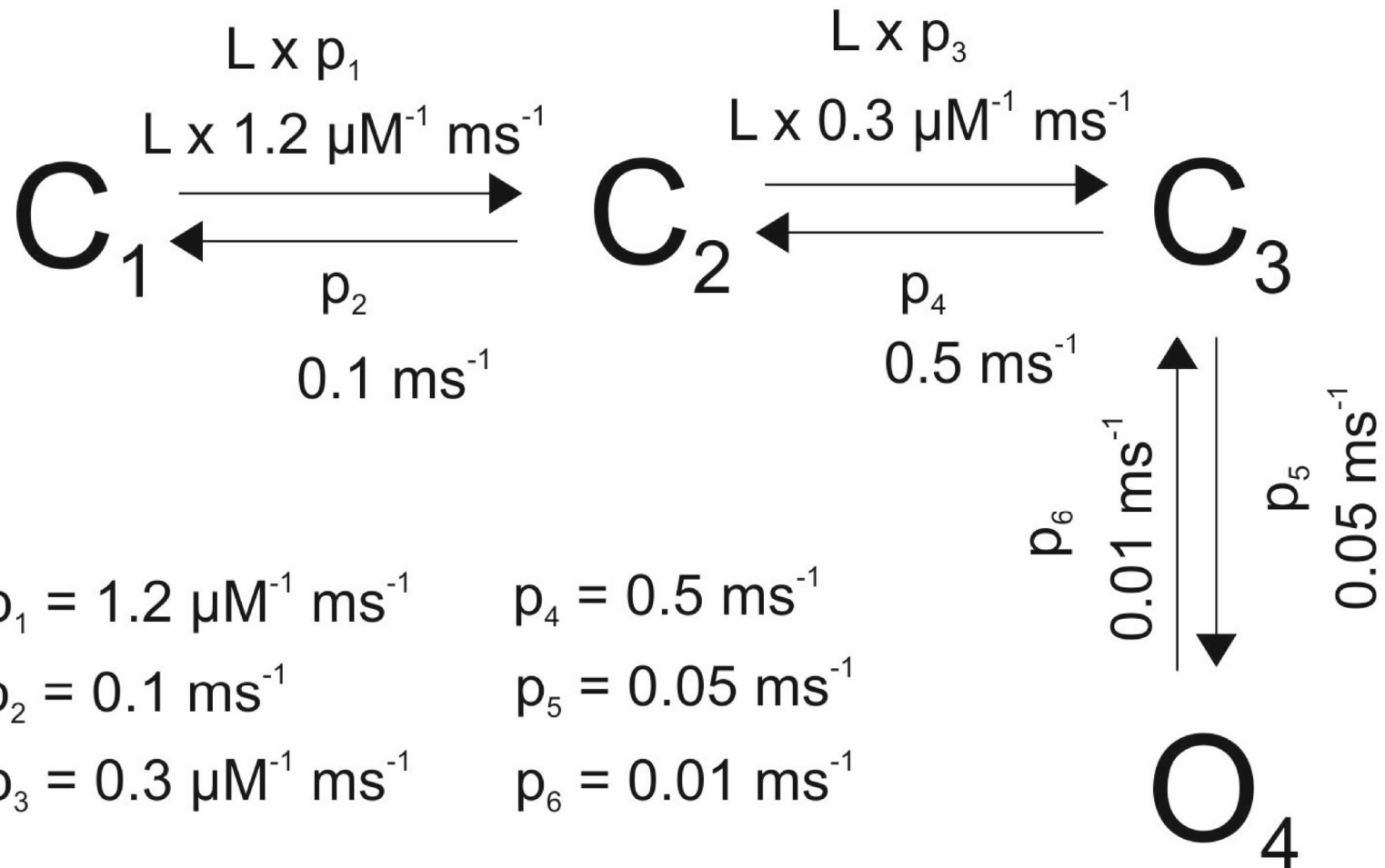

Supplement: Source code 1. [file elife-62714-code1.zip › MakroSim/Modell_3C1O_Jan.pdf]

Modell CCCFFO nach Jan (siehe E-Mail 04.08.21)

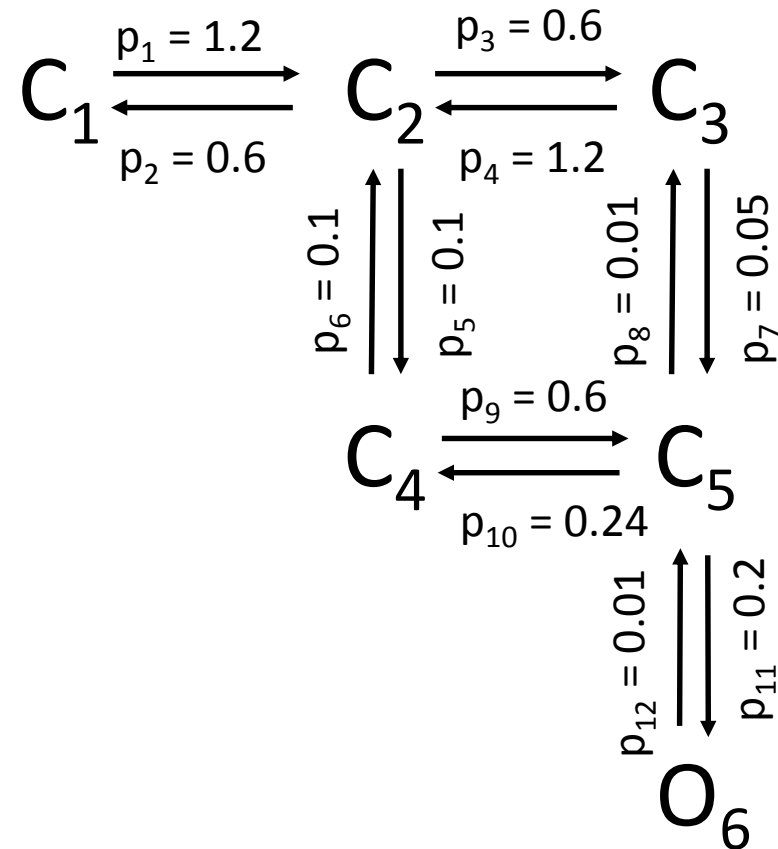

$p_7$  nach Jan 50  
 $p_8$  nach Jan 10  
 $p_{11}$  nach Jan 200  
 $p_{12}$  nach Jan 10

Supplement: Source code 1. [file elife-62714-code1.zip › MakroSim/Modell_CCCFFO.pdf]
